# The role of m6A methylation in osteosarcoma biological processes and its potential clinical value

**DOI:** 10.1186/s40246-022-00384-1

**Published:** 2022-04-18

**Authors:** Yanjiao Wu, Zhiyun Wang, Jianlin Shen, Wei Yan, Shurong Xiang, Huan Liu, Wenhua Huang

**Affiliations:** 1 Shunde Hospital, Southern Medical University (The First People’s Hospital of Shunde), Foshan, China; 2 Guangdong Provincial Key Laboratory of Medical Biomechanics, School of Basic Medical Sciences, Southern Medical University, Guangzhou, China; 3 Guangdong Engineering Research Center for Translation of Medical 3D Printing Application, Southern Medical University, Guangzhou, China; 4 Guangdong Innovation Platform for Translation of 3D Printing Application, Southern Medical University, Guangzhou, China; 5 Department of Orthopaedics, Affiliated Traditional Chinese Medicine Hospital, Southwest Medical University, Luzhou, China; 6 The Precision Medicine Institute, The Third Affiliated Hospital, Southern Medical University, Guangzhou, China; 7 Department of Anatomy, Hebei Medical University, Shijiazhuang, China

**Keywords:** m6A modification, Osteosarcoma, Epigenetics, Writers, Erasers, Readers

## Abstract

Osteosarcoma (OS) is the most common primary malignant bone tumor in children and young adults and has a poor prognosis. Recent developments in the field of high-throughput sequencing technology, particularly in methylated RNA immunoprecipitation sequencing (MeRIP-seq), have led to renewed interest in RNA methylation. Among the various RNA modifications, N6-methyladenosine (m6A) modifications are the most common. Emerging evidence suggests that m6A methylation can affect the complexity of cancer progression by regulating biological functions related to cancer. In this review, we will shed light on recent findings regarding the biological function of m6A methylation in OS and discuss future research directions and potential clinical applications of RNA methyltransferases in OS.

## Background

Osteosarcoma (OS) is the most common primary malignant bone tumor in children and young adults and often occurs in the epiphysis of the long diaphysis [1]. It is derived from stromal cells, and tumor bone-like tissue and bone tissue are formed directly or indirectly through cartilage [2]. The annual incidence rate of OS is estimated at two to four patients per million [3, 4]. OS is the important cause of cancer-related death among children and young adults [5]. The main treatment strategy for OS is neoadjuvant chemotherapy combine with surgical resection of the primary tumor and subsequent adjuvant chemotherapy [6]. However, due to delayed diagnosis, metastasis and recurrence, the 5-year overall survival rate is only approximately 20% [7]. Thus, it is imperative to understand the underlying molecular mechanism of occurrence, development, metastasis and prognosis of OS.

Previous studies have found that epigenetic modifications play a key role in the occurrence and development of OS. Epigenetic modifications include chemical modifications of DNA, RNA and protein [8], which are characterized by changes in gene expression and function without changes in gene sequence [9]. RNA modifications are abundant, diverse and ubiquitous. RNA modifications can regulate a variety of molecular processes including RNA splicing, translation, localization, binding to proteins or other RNAs [10, 11]. RNA methylation is a posttranscriptional modification that exists in all organisms. It is closely related to important biological processes and thus to many human diseases [12, 13]. Over the past decade, RNA methylation has been a popular topic of biomedical research. N6‐methyladenosine (m6A) modification is the most commonly studied RNA modification. Current studies have shown that m6A methylation plays critical roles in the pathogenesis of many cancers, including lung cancer [14], liver cancer [15] and gastric cancer [16]. Similarly, the functions of m6A are critical for tumor initiation, promotion, and progression in OS. This paper reviews the relationship between m6A methylation and OS: m6A methylation is involved in the occurrence, development, metastasis and prognosis of OS. We also discuss the potential clinical applications and future directions of m6A modification as a biomarker as well as a therapeutic target of OS. The information presented here provides new ideas for the treatment of OS. It will help better treat patients with OS in future.

## m6A methylation

m6A methylation was first discovered in 1974 [17]. It is the most abundant internal modification of RNA in eukaryotic cells, accounting for more than 80% of all RNA modifications [18]. In recent years, with the rapid development of high-throughput and high-sensitivity sequencing methods, the universality and importance of m6A modification have gradually been recognized [19, 20]. m6A has a conserved modified gene sequence, which is distributed and enriched in the long exon, near the stop codon and 3′ untranslated regions (UTRs). m6A modification can transfer a methyl to the N-6 position of the adenosine in the nucleic acid [14, 21]. As a transcriptome regulator of gene expression, m6A modification can affect pre-mRNA splicing and mRNA transport, degradation and translation [22]. The process of m6A methylation is dynamic and reversible and is regulated by m6A methylation regulators ("writers", "erasers" and "readers") [23].

### m6A writers

m6A writers are multicomponent methyltransferase complexes. Known components of such complexes are methyltransferase-like 3 (METTL3), methyltransferase-like 14 (METTL14), methyltransferase-like 16 (METTL16), Wilm’s tumor-associated protein (WTAP), zinc-finger CCCH domain-containing protein 13 (ZC3H13), vir-like m6A methyltransferase-associated (VIRMA, also called KIAA1429), RNA-binding motif protein 15 (RBM15/15B), etc. [24–26]. METTL3 was the first m6A writer protein to be identified. It is the most important component of the methyltransferase complex (MTC) but has no enzyme activity alone without METTLE4 [27–30]. In the MTC, METTL3 and METTL14 can form a stable METTL3-METTL14 complex at a ratio of 1:1 [31]. METTL3 is the active catalyzing enzyme, while METTL14 can enhance the activity of METTL3 and stabilize the structure of the MTC [30]. In addition, METTL14 is responsible for promoting substrate binding. WTAP can bind to the METTL3-14 complex and participate in catalytic activity and element localization in nuclear speckles [32]. KIAA1429 can mediate mRNA m6A methylation in 3′UTRs and near stop codons [33]

### m6A erasers

m6A erasers are demethylases that can mediate reversible and dynamic m6A modification. Fat mass and obesity-associated protein (FTO) were the first m6A demethylase to be discovered [34]. FTO oxidizes m6A into N6-hydroxymethyladenosine and N6-formyladenosine and demethylates m6A both in vivo and in vitro [35]. The α-ketoglutarate-dependent dioxygenase alk B homolog 5 (ALKBH5) was the second m6A demethylase to be identified (in 2013) and is localized to the nucleus [36]. ALKBH5, as an FTO homolog but different from FTO, directly catalyzes modification of m6A to adenosine without a detected intermediate [36].

### m6A readers

The regulation of mRNA processing and metabolism by m6A largely depends on the effective recognition of m6A binding proteins. These binding proteins are called m6A readers. The YT521-B homology (YTH) domain family includes YTHDF1, YTHDF2, YTHDF3, YTHDC1, YTHDC2, which can recognize m6A modifications and regulate multiple biological functions [37]. YTHDC1 is localized to the nucleus and can regulate RNA alternative splicing and nuclear export [38, 39]. Other family members are located in the cytoplasm and can identify specific m6A sites to exert posttranscriptional functions [40–43]. Other m6A readers, such as ELAV-like protein 1 (ELAVL1) [44], insulin-like growth factors (IGF2BP1, IGF2BP2 and IGF2BP3) [45–47] and heterogeneous nuclear ribonucleoproteins (HNRNPC and HNRNPA2/B1) [48, 49], have been identified.

## The role of m6A methylation in OS

Recently, emerging evidence has revealed that m6A methylation is closely associated with processes related to the progression of OS, including tumor proliferation, apoptosis, migration, invasion, metastasis and drug resistance. In this section, we review the recent findings related to m6A methylation in OS (Table 1).

**Table 1:** Role of m6A modulators in osteosarcoma

m6A regulators	Function	Target	Upstream	Role in	Biological function	Mechanism	Vitro/vivo	References
METTL3	Writers	ATAD2	–	Oncogene	Promotes cell proliferation, invasion and migration; Inhibits apoptosis	–	Vitro	[51]
METTL3	Writers	LEF1	–	Oncogene	Promotes cell proliferation, invasion, migration and tumor growth	Activation of the wnt/β-catenin signaling pathway	Vitro and Vivo	[52]
METTL3	Writers	DRG1	–	Oncogene	Promotes cell migration and colony formation; Inhibits apoptosis	–	Vitro	[53]
METTL3	Writers	TRIM7	–	Oncogene	Promotes cell invasion and migration; Unfavorable response to chemotherapy	–	Vitro and Vivo	[59]
METTL14	Writers	TRIM7	–	Oncogene	Promotes cell invasion and migration; Unfavorable response to chemotherapy	–	Vitro and Vivo	[59]
WTAP	Writers	HMBOX1	–	Oncogene	Promotes cell proliferation, invasion and migration	Activation of the PI3K/AKT signaling pathway	Vitro and Vivo	[55]
KIAA1429	Writers	–	miR-143-3p	Oncogene	Promotes cell proliferation, invasion and migration, tumor growth; Unfavorable response to chemotherapy	Activation of the Notch signaling pathway	Vitro and Vivo	[56]
ALKBH5	Erasers	PVT1	–	Oncogene	Promotes cell proliferation and tumor growth	–	Vitro and Vivo	[54]
ALKBH5	Erasers	YAP	–	Tumor suppressor	Suppresses cell proliferation, invasion and migration; Trigger cell apoptosis	Activation of pre-miR-181b-1/YAP signaling	Vitro and Vivo	[72]
YTHDF2	Readers	–	miR-766 circ_0001105	Tumor suppressor	Suppresses cell proliferation, invasion and migration; Favorable response to chemotherapy	–	Vitro and Vivo	[57]
YTHDF2	Readers	TRIM7	–	Oncogene	Promotes cell invasion and migration; Unfavorable response to chemotherapy	–	Vitro and Vivo	[59]
ELAVL1	Readers	DRG1	–	Oncogene	Promotes cell migration and colony formation; Inhibits apoptosis	–	Vitro	[53]

### The role of m6A methylation in the proliferation and apoptosis of OS

Deregulation of cell proliferation and suppression of cell death together promote the progression of cancer [50]. Researchers have found that the m6A writer METTL3 plays a role as an oncogene in the progression of OS and is located in the cytoplasm and nucleus of OS cells. Zhou et al. [51] found that silencing METTL3 in SAOS-2 and MG63 cells significantly inhibited the m6A methylation level, inhibited cell proliferation and promoted cell apoptosis. However, the proliferation and apoptosis of U2OS cells was not significantly affected by METTL3 overexpression. Further mechanistic analysis suggested that METTL3 promotes cell proliferation and inhibits apoptosis in OS cells by regulating the expression of ATPase family AAA domain containing 2 (ATAD2). In another study, METTL3 silencing inhibited the proliferation of HOS and SAOS-2 cells by regulating the m6A level of LEF1 and activating the Wnt/β-catenin signaling pathway [52]. Another study also showed that developmentally regulated GTP-binding protein 1 (DRG1) acts as an oncogene and mediates cell viability, cell cycle distribution and apoptosis in OS cells. METTL3 knockdown inhibited the viability of OS cells, arrested the cell cycle in the G2/M stage and induced apoptosis by decreasing the m6A and mRNA levels of DRG1 [53]. In addition to METTL3, other m6A modulators can also regulate proliferation and apoptosis of OS cells in vitro and in vivo. Knockdown of ELAVL1 also inhibited proliferation and induced apoptosis by impairing the stability of DRG1 mRNA [53]. Plasma variant translocation 1 (PVT1) is a well-known oncogenic long noncoding RNA (lncRNA). The m6A demethylase ALKBH5 can bind to PVT1, inhibit its degradation and reduce m6A modification of PVT1. The upregulation of PVT1 mediated by ALKBH5 promotes proliferation in vitro and tumor growth in vivo [54]. WTAP, as an m6A writer, was found to be involved in the proliferation of OS in vitro and in vivo [55]. CCK-8 and colony formation assays showed that silencing WTAP significantly repressed the proliferative capacity of OS cells in vitro. In subcutaneous OS mice, WTAP deficiency significantly reduces tumor size and tumor weight. A previous study demonstrated that silencing KIAA1429 could reduce OS cell proliferation in vitro, as well as tumor growth in vivo [56]. In OS cells, YTHDF2 significantly suppresses proliferation by regulating miR-766 [57]. In summary, these findings reveal that m6A is essential for the proliferation and apoptosis of OS cells in vitro and in vivo.

### The role of m6A methylation in the migration, invasion and metastasis of OS cells

Tumor cell migration and invasion are critical factors for tumor progression and metastasis. Tumor metastasis remains the number one cause of cancer-related death [58]. Multiple studies have shown that METTL3 is associated with the migration, invasion and metastasis of OS cells. A previous study showed that silencing METTL3 in SAOS-2 and MG63 cells dramatically inhibited migration and invasion. However, overexpression of METTL3 had no significant effect on the migration and invasion of U2OS cells [51]. Another study suggested that METTL3 silencing significantly repressed the migration and invasion of HOS and SAOS-2 cells. Compared with the control group, the METTL3 silencing group exhibited decreased progression of bone tumors in vivo [52]. In HOS and U2OS cells, wound healing assays showed that silencing WTAP significantly reduces the migration ability of OS cells. Transwell invasion assays suggested that silencing WTAP represses migration [55]. In vitro migration and invasion assays were performed, and the results indicated that the invasion and migration of OS cells are significantly reduced by KIAA1429 knockdown [56]. m6A methylation may affect the migration and invasion of OS cells through indirect regulation of the stability, degradation and maturation of mRNAs or noncoding RNAs. In U2OS and MG63 cells, ectopic overexpression of YTHDF2 significantly suppressed OS cell invasion by regulating miR-766 [57]. In HOS and MG63 cells, downregulation of TRIM7 significantly repressed cell invasion and migration. Silencing the m6A reader YTHDF2 significantly increased the mRNA level of TRIM7. METTL3 and METTL14 can promote the m6A modification of TRIM7 in OS cells [59]. DRG1 knockdown was directly associated with the suppression of migration but did not modify the effect on cell invasion. Knockdown of METTL3 and ELAVL1 impaired the m6A modification and expression level of DRG1 [53]. Collectively, these findings reveal that RNA methyltransferases play an important role in the migration, invasion and metastasis of OS cells.

## Potential clinical application of m6A methylation in OS

An increasing number of studies have shown that m6A modulators are closely related to the clinical features of patients with OS. The abnormal expression of m6A-related regulatory factors in OS is closely related to poor prognosis and chemotherapy resistance of OS. m6A modification may serve as a novel prognostic diagnostic biomarker or potential therapeutic target for OS (Table 2).

**Table 2:** Potential clinical application of m6A methylation in osteosarcoma

Source	Non-tumor samples	Tumor samples	m6A regulators	Role in	Potential application	References
Publicly datasets	17	306	METTL3, KIAA1429, HNRNPA2B1, FTO, METTL14, YTHDF2	Poor prognosis	Biomarker	[60]
Tissue microarray	65	120	METTL3, KIAA1429, HNRNPA2B1, FTO, METTL14, YTHDF2	Poor prognosis	Biomarker	[60]
Publicly datasets	80	80	METTL3 and ALKBH5	Poor prognosis	Biomarker	[61]
Clinical samples	70	70	ALKBH5	Poor prognosis Oncogene	Biomarker Therapeutic target	[54]
Publicly datasets	3	44	WTAP	Poor prognosis Oncogene	Biomarker Therapeutic target	[55]
Clinical samples	104	104	WTAP	Poor prognosis Oncogene	Biomarker Therapeutic target	[55]
Tissue microarray	65	120	KIAA1429	Poor prognosis Chemotherapy resistance	Biomarker Therapeutic target	[56]
Tissue microarray	65	120	YTHDF2	Chemotherapy resistance	Therapeutic target	[57]

### m6A methylation is associated with poor prognosis of OS

The expression of m6A-related regulatory factors was comprehensively analyzed in OS and normal tissues. In a tissue microarray (TMA) cohort, high expression of METTL3, KIAA1429 and HNRNPA2B1 and low expression of FTO, METTL14 and YTHDF2 were prognostic markers for poor clinical outcomes in OS [60]. A study explored the relationship between m6A-related regulatory factor expression in biopsy specimens and the metastasis-free survival rate in 88 OS patients. High expression of METTL3 and ALKBH5 showed a tendency to be associated with poor prognosis in OS [61]. Chen et al. proposed that ALKBH5 mRNA levels were significantly upregulated in OS tissues compared to adjacent normal tissues. High ALKBH5 expression was associated with poor overall survival in patients with OS [54]. Chen et al. verified that significantly higher mRNA and protein levels of WTAP were present in OS tissues than in adjacent normal tissues. High WTAP expression in patients with OS has been associated with tumor size, metastasis and TNM stage, and overexpression of WTAP has been correlated with poor prognosis [55]. KIAA1429 mRNA expression was markedly higher in OS tissues than in adjacent normal tissues. KIAA1429 overexpression was related to unfavorable prognosis in OS [56].

### The role of m6A methylation in chemotherapy and radiotherapy resistance in OS

Surgery combined with chemotherapy and radiotherapy is the most commonly used treatment for advanced tumors [62]. However, resistance to radiotherapy and chemotherapy leads to disease recurrence and therapeutic failure [63]. Researchers have thoroughly analyzed m6A methylation in the transcriptome of OS cells after chemotherapy, revealing that m6A is an important part of posttranscriptional regulation. m6A methylation changes occur in OS cells after chemotherapy [61]. To explore the role of the TRIM7 response to chemotherapy in OS, MG63 and SAOS-2 OS cells with high TRIM7 expression or low TRIM7 expression were transplanted into mice. After adriamycin or methotrexate chemotherapy, tumors with high TRIM7 expression were larger than those with low TRIM7 expression. TRIM7 upregulation was induced by m6A modification in a METTL3/14-YTHDF2-mRNA decay-dependent manner and promoted OS chemoresistance [59]. An analysis of the relationship between the YTHDF2 expression level and clinicopathological characteristics was conducted by using a TMA cohort. The results showed that low expression of YTHDF2 in OS tissues was significantly associated with poor response to chemotherapy [57]. Clinical analysis of TMA data from 120 OS patients in public databases showed that high KIAA1429 expression was closely associated with chemotherapy resistance in OS [56]. A recent study indicated that m6A methylation plays an important role in the ultraviolet-induced DNA damage response. In OS cells, low METTL3 expression leads to delayed repair of ultraviolet-induced cyclobutane pyrimidine dimers and increases sensitivity to irradiation [64]. These observations suggest that RNA methyltransferases are involved in chemoradiotherapy resistance in OS, indicating that RNA methyltransferases may be potential targets for reversing chemoradiotherapy resistance.

## Discussion

**Fig. 1:**
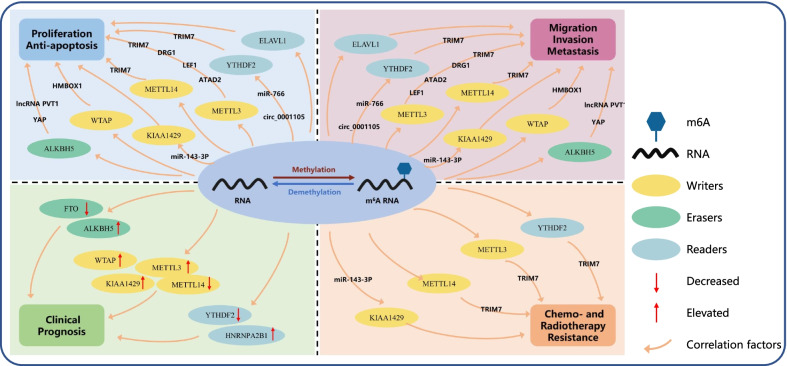
The potential roles of RNA m6A modification in osteosarcoma. m6A regulates the differential expression of oncogenes and tumor suppressor genes, which contributes to processes involved in the development of osteosarcoma, including cell proliferation, apoptosis, invasion, migration, metastasis, clinical prognosis, chemotherapy and radiotherapy resistance

m6A methylation is an emerging research field. A number of studies have proven that the m6A modification of RNA plays an important role in the occurrence, development, metastasis and prognosis of multiple cancer types [65–71]. In this review, we summarized recent advances in the understanding of the role of m6A methylation in OS biological processes and its potential clinical value (Fig. 1). It is important to note that the mechanism of m6A methylation in OS is complex and even inconsistent. Chen et al. showed that ALKBH5 mRNA levels were significantly upregulated in OS tissues compared to adjacent normal tissues. High ALKBH5 expression was associated with poor overall survival in patients with OS [54]. In contrast, Yuan et al. reported that ALKBH5 suppresses OS progression via m6A-dependent epigenetic silencing of the pre-miR-181b-1/YAP signaling axis [72]. m6A methylation, as a double-edged sword, is also commonly seen in other types of tumors [65, 73]. In colorectal cancer, Li et al. [74] showed that METTL3 promotes cancer progression, while Deng et al. [75] reported that METTL3 suppresses cancer progression. There are many potential reasons for this phenomenon, including but not limited to: (1) The samples and methods used in the study are different; (2) The origin of tumor tissue is different; (3) Tumor heterogeneity. More convincing studies are needed to further explore the regulatory mechanism of m6A in different tumors.

RNA methylation has high tissue specificity in human body [76]. m6A sequencing results of nine tissues in adults showed that more than 36.7% of m6A sites were found in only one specifc tissue, and only 5.5% of the sites were shared in all tissues [77]. The tissue specificity of RNA methylation may be related to cancer metastasis. Compared with primary breast cancer, the expression of YTHDF3 was significantly increased in breast brain metastase, but there was no change in lung, bone, liver, spleen, lymph nodes and adrenal metastases. Further experiments have shown that over expression of YTHDF3 is a key step in the brain metastasis of breast cancer [42]. Some m6A regulators have been shown to promote the metastasis of OS. However, whether the tissue specificity of RNA methylation can affect the direction of OS metastasis that has not been studied. It provides a new perspective for us to study the metastasis of OS.

Although the understanding of the roles of m6A in OS has markedly advanced in recent years, many challenges remain. First, the mechanisms of m6A methylation in OS are largely unknown. Second, many studies have suggested that the m6A level and m6A regulators had the potential to be diagnostic and prognostic biomarkers for OS, but the specificity and sensitivity of these biomarkers need to be explored in large patient cohorts. Third, prior studies have noted the potential of regulators and related pathways as therapeutic targets in OS. Most studies have focused on the molecular mechanisms of m6A regulators but lack drug development and preclinical/clinical studies. In addition, possible side effects should also be investigated with further detailed studies.

## Conclusions

m6A methylation has been a hot research topic in recent years, but related research in OS is still in its infancy. More m6A methylation associated with OS will be identified in future using high-throughput sequencing technology, which will screen out more candidate diagnosis and prognosis biomarkers of OS. In clinical application, some certain methylation alterations detection for monogenic or polygenic will be used detected biomarkers levels in OS patients. It is of great significance to find potential therapeutic targets and tumor markers for OS and improve the status quo of OS treatment.

## Data Availability

Not applicable.

## Abbreviations

OS: Osteosarcoma
m6A: N6-methyladenosine
MeRIP-seq: Methylated RNA immunoprecipitation sequencing
METTL3: Methyltransferase like 3
METTL14: Methyltransferase like 14
METTL16: Methyltransferase like 16
WTAP: Wilm’s tumor-associated protein
ZC3H13: Zinc finger CCCH domain containing protein 13
VIRMA: Vir-like m6A methyltransferase associated
RBM15: RNA-binding motif protein 15
MTC: Methyltransferase complex
FTO: Fat mass and obesityassociated protein
ALKBH5: α-Ketoglutaratedependent dioxygenase alk B homolog 5
YTH: YT521-B homology
YTHDC1: YTH domain-containing protein 1
YTHDF1: YTH domain-containing family protein 1
YTHDF2: YTH domain-containing family protein 2
YTHDF3: YTH domain-containing family protein 3
YTHDC2: YTH domain-containing protein 2
ELAVL1: ELAV-like protein 1
IGF: Insulin-like growth factor
HNRNP: Heterogeneous nuclear ribonucleoprotein
ATAD2: ATPase family AAA domain containing 2
DRG1: GTP-binding protein 1
PVT1: Plasma variant translocation 1
lncRNA: Long noncoding RNA
